# Restitution Slope Affects the Outcome of Dominant Frequency Ablation in Persistent Atrial Fibrillation: CUVIA-AF2 *Post-Hoc* Analysis Based on Computational Modeling Study

**DOI:** 10.3389/fcvm.2022.838646

**Published:** 2022-03-03

**Authors:** Je-Wook Park, Byounghyun Lim, Inseok Hwang, Oh-Seok Kwon, Hee Tae Yu, Tae-Hoon Kim, Jae-Sun Uhm, Boyoung Joung, Moon-Hyoung Lee, Hui-Nam Pak

**Affiliations:** Division of Cardiology, Department of Internal Medicine, Yonsei University College of Medicine, Yonsei University Health System, Seoul, Republic of Korea

**Keywords:** atrial fibrillation, computational modeling, dominant frequency, restitution, recurrence

## Abstract

**Introduction:**

Although the dominant frequency (DF) localizes the reentrant drivers and the maximal slope of the action potential duration (APD) restitution curve (Smax) reflects the tendency of the wave-break, their interaction has never been studied. We hypothesized that DF ablation has different effects on atrial fibrillation (AF) depending on Smax.

**Methods:**

We studied the DF and Smax in 25 realistic human persistent AF model samples (68% male, 60 ± 10 years old). Virtual AF was induced by ramp pacing measuring Smax, followed by spatiotemporal DF evaluation for 34 s. We assessed the DF ablation effect depending on Smax in both computational modeling and a previous clinical trial, CUVIA-AF (170 patients with persistent AF, 70.6% male, 60 ± 11 years old).

**Results:**

Mean DF had an inverse relationship with Smax regardless of AF acquisition timing (*p* < 0.001). Virtual DF ablations increased the defragmentation rate compared to pulmonary vein isolation (PVI) alone (*p* = 0.015), especially at Smax <1 (61.5 vs. 7.7%, *p* = 0.011). In post-DF ablation defragmentation episodes, DF was significantly higher (*p* = 0.002), and Smax was lower (*p* = 0.003) than in episodes without defragmentation. In the *post-hoc* analysis of CUVIA-AF2, we replicated the inverse relationship between Smax and DF (*r* = −0.47, *p* < 0.001), and we observed better rhythm outcomes of clinical DF ablations in addition to a PVI than of empirical PVI at Smax <1 [hazard ratio 0.45, 95% CI (0.22–0.89), *p* = 0.022; log-rank *p* = 0.021] but not at ≥ 1 (log-rank *p* = 0.177).

**Conclusion:**

We found an inverse relationship between DF and Smax and the outcome of DF ablation after PVI was superior at the condition with Smax <1 in both *in-silico* and clinical trials.

## Introduction

Atrial fibrillation catheter ablation (AFCA) is a modality for atrial fibrillation (AF) rhythm control and has a beneficial effect on heart failure mortality and heart failure admission (1, 2). Nevertheless, it is difficult to maintain long-lasting sinus rhythm after AFCA in especially patients with persistent AF (3). Extrapulmonary vein (PV) foci, and PV, can have an essential role in maintaining long-lasting sinus rhythm after the AFCA (4). However, additional ablation, including linear ablation and complex fractionated electrograms (CFAE) ablation after circumferential PV isolation (CPVI), did not improve ablation outcomes (5). Based on personalized pathophysiology of AF, ablation for targeting AF drivers improved the rhythm outcome of AFCA (6). However, in the clinical setting, there was a controversial result of AFCA for targeting AF drivers (7). In addition, it might be difficult to determine the exact location of AF drivers in the clinical AF mapping system (8). Ablation for targeting AF drivers, which were visualized and located precisely by computational simulation, might improve the ablation outcome of AFCA.

We previously reported that AF drivers were well localized by dominant frequency (DF), and ablation for AF drivers localized by DF showed better rhythm outcomes in computational simulation (9, 10). During the clinical ablation based on a computational simulation, Boyle et al. showed the possibility and feasibility of a clinical AF ablation targeting computationally detected reentrant drivers using MRI (11). Further, Baek et al. reported that a computational-guided DF ablation could benefit the clinical AF rhythm outcomes (12). However, it has not been well established whether ablation of clinically detected AF drivers has a beneficial effect on rhythm outcomes (13–16). Kim et al. reported that the maximal slope of the action potential duration (APD) restitution curve (Smax) was related to the perpetuation of AF (17). The APD restitution reflects the dynamic heterogeneity of the APD, and a continuous wave-break is easily maintained under a condition with a high Smax and large change in the APD in response to a constant diastolic interval (DI) change (18–21). The slope of the APD restitution curve consists of the APD as the Y-axis and DI as the X-axis in the coordinate plot (Supplementary Figure 1). The preceding DI determines the following APD in the APD restitution curve (20, 21). Especially, when the slope of the APD restitution curve is steep (>1), a small change in the DI results in a large change in the APD as compared to a low slope of the APD restitution curve (20, 21). When this oscillation of the APD becomes large enough, the differences in the refractoriness between adjacent cardiomyocytes result in a local partial conduction block leading to wave-break at some point of the reentrant wavefronts (18–21). Therefore, AF maintenance could consist of several mechanisms instead of a single pathway (22). However, the direct relationship between Smax and DF has not yet been known. In this study, we hypothesized that DF ablation has different effects on AF depending on Smax. We investigated the relationship between Smax and DF and the rhythm outcomes of DF ablation depending on the value of Smax in computational simulation and clinical patients with AF.

## Methods

### Study Population

This study protocol adhered to the principles of the Declaration of Helsinki and was approved by the Institutional Review Board of the Yonsei University Health System. All patients provided written informed consent for inclusion in the Yonsei Cohort Database (ClinicalTrials.gov Identifier: NCT02138695). Twenty-five patients (68% male, 60 ± 10 years old, 32% paroxysmal AF) who underwent AFCA in the Yonsei AF Cohort were included in this study for computational modeling. We evaluated the clinical role of wave dynamics in 170 clinical patients (70.6% male, 60 ± 11 years old, mean 13 ± 6.5 months of follow-up) from the previous randomized clinical trial CUVIA AF2 database (12) who underwent AFCA for persistent AF. The exclusion criteria of CUVIA AF2 were as follows: (1) age younger than 20 or older than 80 years, (2) paroxysmal AF, (3) valvular AF, (4) significant structural heart disease other than left ventricular hypertrophy, (5) left atrial (LA) diameter > 55 mm, (6) previous history of AF ablation or cardiac surgery, and (7) an LA voltage map was not available due to recurrent (>3 episodes) or reinitiated AF after cardioversion.

### Computational Modeling of AF

**Figure 1:**
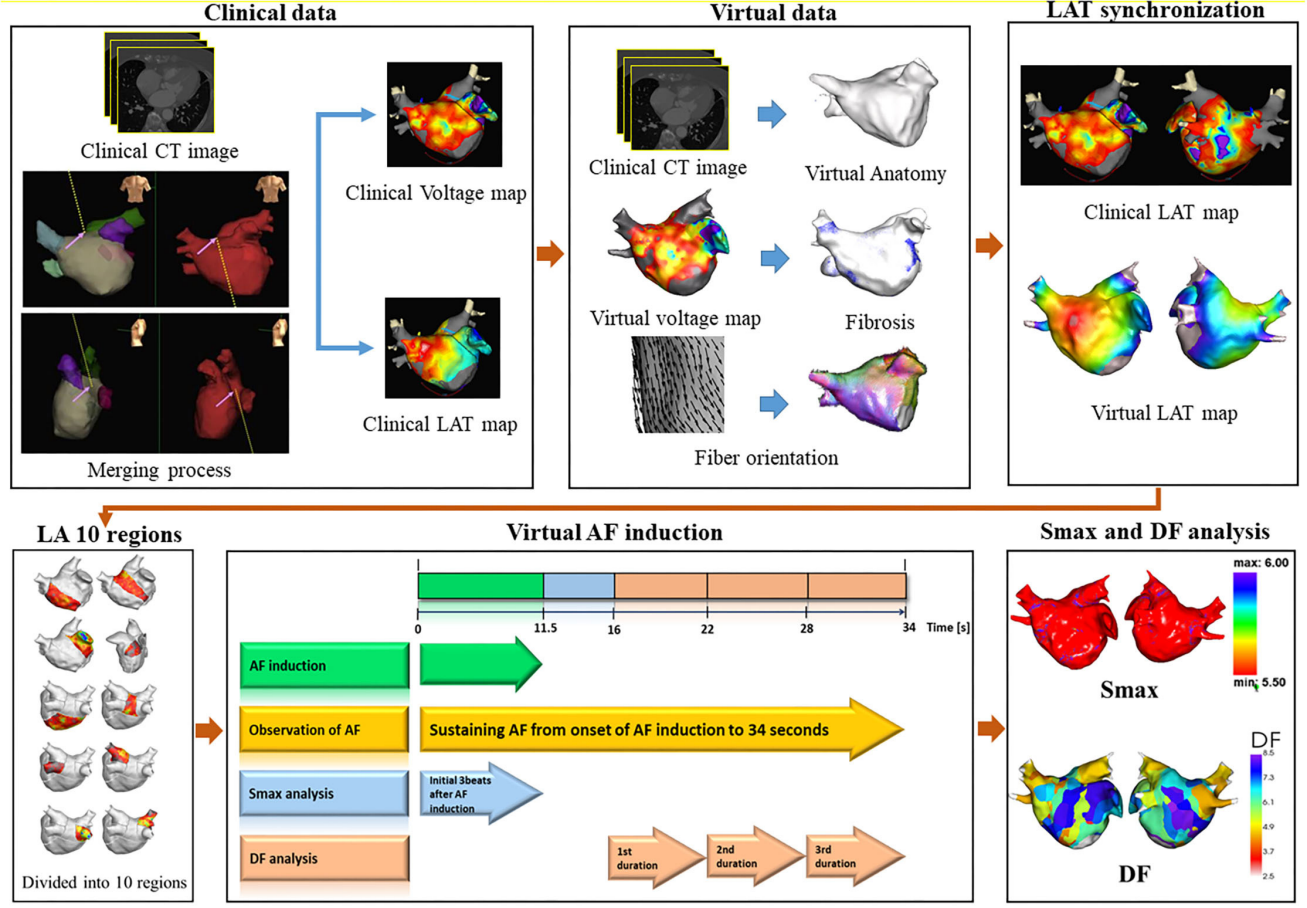
Study protocol of computational atrial modeling. CT, computed tomography; LAT, local activation time; LA, left atrium; AF, atrial fibrillation; Smax, the maximal slope of action potential restitution curve; DF, dominant frequency.

To develop a computational AF modeling reflecting the individual atrial anatomy, tissue characteristics, and electrophysiology, we integrated the clinical electroanatomical map (voltage and local activation time maps) acquired during sinus rhythm into the computational modeling software (CUVIA, Model:SH01; Laonmed Incorporation, Seoul, Korea) to develop realistic atrial modeling (23). Figure 1 shows the summary of the computational atrial modeling in this study.

#### Electroanatomical Mapping Merged With CT Images

Merging of the electroanatomical mapping with the CT images was conducted by four consecutive steps: geometry, trimming, field scaling, and alignment steps. To acquire the clinical LA voltage map and local activation map, a clinical electroanatomical map merged with the CT image was obtained during the clinical ablation procedure in each patient. Using an Ensite NavX system (Abbott Inc., Lake Bluff, Illinois, USA), we obtained the clinical LA voltage data during sinus rhythm based on the bipolar electrograms recorded from about 500 points on the LA during the AFCA. Using the method embedded in the Ensite NaVx system, the technician coordinated the 3D LA modeling results with the clinical map after merging it with the cardiac CT images of the patient. Furthermore, we also stored the LA voltage data from each clinical catheter point during the procedure.

#### Developing a Computational 3D Mesh Model Derived From the CT Image and Ionic Remodeling

In the computational simulation lab, using the CUVIA software (23), a 3D mesh model of the LA geometry was developed based on the CT images, and the mesh of the LA surface was refined as a triangular shape. About 400,000 nodes were developed in this model. The distance between adjacent nodes was about 300 μm. We used the human atrial myocyte model developed by Courtemanche et al. (24) for the ionic currents of each cell. For the ionic remodeling of AF, the IK1 and INCX were increased by 100 and 40%, respectively, and the INa, Ito, ICaL, and IKur were decreased by 10, 70, 50, and 50%, respectively (25).

#### Developing the Fiber Orientation and Virtual Voltage Map

We used the atlas-based method (26) to estimate a personalized fiber orientation, which reflected the anisotropic conduction flow from the isotropic triangular mesh. The GPU-based fiber tracking process was performed with two steps: tracking and visualization. Fiber tracking involved a parallel task including GPU-based fiber tracking and visualization of the fiber orientation on the 3D-local activation time map. We determined the vector of the fiber orientation at each node on the 3D mesh model according to the myocardial fiber direction. The fiber tracking method determined the difference in the conduction based on the fiber orientation (23). The conduction velocity in the same direction as the fiber vector was faster than that in the perpendicular direction to the fiber vector.

The virtual voltage data were developed through interpolation of the clinical bipolar voltage data. The inverse distance weighting (IDW) method (27) was used to interpolate the clinical voltage data for the virtual voltage data. The detailed equation for the IDW was as follows:


$$\begin{array}{l} W_{ij}\;=\;\frac{{d_{ij}}^{-a}}{\sum_{k}^{n_{j}}{d_{k}}_{j}},\;R_{j}\;=\;\sum_{i\;=\;1}^{n_{j}}w_{ij}R_{ij} \end{array}$$

where *W* indicated the weighted average of the neighboring values; *i* and *j* indicated the unknown and known values of the respective points; $d_{ij}^{-\text{a}}$ was the distance between the unknown and known points; *R*_*j*_ indicated the interpolation value at the unknown point *j*; and *R*_*ij*_ indicated the known point of the value. The interpolation process produced the virtual voltage data with an amplitude within a 10-mm radius from the region of interest.

#### Developing the Spatial Distribution of the Fibrosis and Conduction Velocity

The fibrosis area was determined based on a clinically obtained bipolar voltage map. The clinical bipolar voltage data was interpolated into the nodes on the computational 3D LA model. After that, we obtained each node's virtual voltage data to determine the fibrosis of the computational 3D LA model. To assess the fibrosis status (yes/no) for each node, we used the following equation (28):


$$\begin{array}{l} P_{\text{fibrosis}}=\\ \{\begin{array}{cc} 1, & X=0\\ -40.0X^{3}+155X^{2}-206X+99.8, & 0<X\leq 1.74\\ 0, & 1.74<X \end{array} \end{array}$$

where *X* indicated the virtual voltage at each node, with a range from 0 to 1.74 mV. If *X* was >1.74 mV, then *P*_fibrosis_ would be zero. Based on the fiber orientation and fibrosis map, the conductivity values of our model (29) were 0.1264 S/m (nonfibrotic longitudinal cell), 0.0546 S/m (fibrotic longitudinal cell), 0.0252 S/m (nonfibrotic longitudinal cells), and 0.0068 S/m (fibrotic longitudinal cell).

#### Synchronization Between the Clinical and Virtual Local Activation Time

The virtual local activation time (LAT) map was synchronized with the individual clinical local activation time map. Before a preliminary simulation, the conduction velocity was calculated by measuring the distance and travel time from the pacing site to the LA appendage. The conduction velocity of the clinical and virtual LAT maps was matched by modulating the diffusion coefficient (23). After that, a color scale indicating the conduction time was examined to achieve a realistic conduction environment. Most conduction velocities were adjusted appropriately when the voltage map-based fibrosis was applied at the default conductivity (29). If a difference from the LAT map still existed, we delicately adjusted the diffusion coefficient, which reflected the node to node conductivity, to synchronize the clinical and virtual LAT maps.

### Protocol of AF Induction and Analysis for Smax and DF

**Figure 2:**
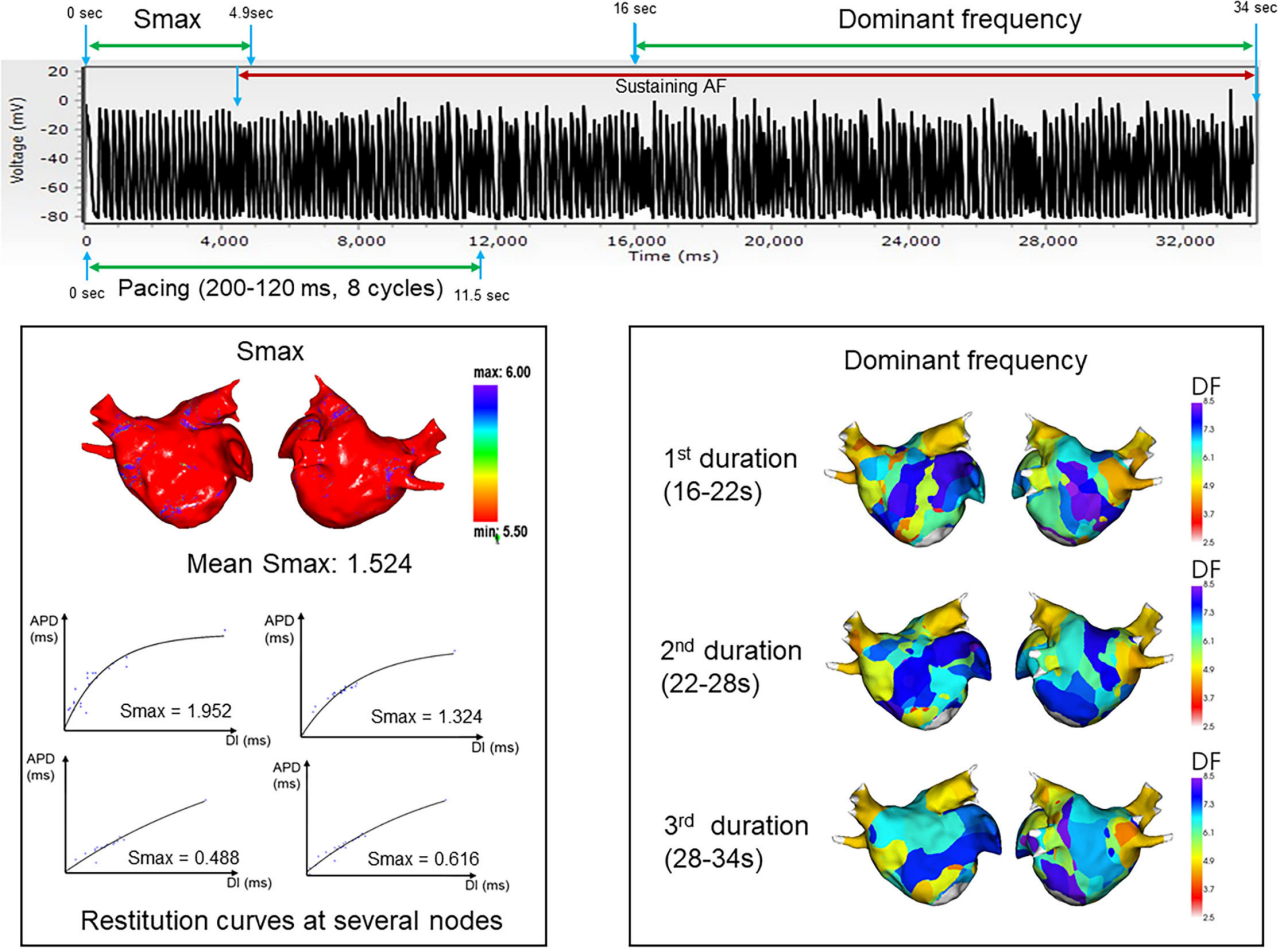
Summary and example of how the Smax and dominant frequency were measured during the virtually induced atrial fibrillation. Smax, the maximal slope of action potential restitution curve; DF, dominant frequency.

**Figure 3:**
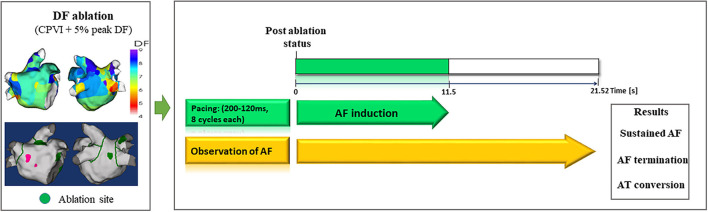
Study protocol of computational virtual DF ablation and AF induction on postablation status. DF, dominant frequency; AF, atrial fibrillation; AT, atrial tachyarrhythmia.

Figures 2, 3 represent the simulation protocol in this study. The CUVIA software (Model: SH01; Laonmed Incorporation, Seoul, Korea) was utilized to conduct virtual AF induction and analysis of wave dynamics including DF and Smax, and perform virtual DF ablation. To induce virtual AF, the ramp-pacing stimulation was initiated on the anterior side of the LA and was conducted with eight cycles of lengths of 200–120 ms (10). The duration of the pacing was set from 0 to 11.52 s to induce AF. If AF was induced within 11.52 s, we observed the maintenance of AF from the onset of AF induction to 34 s. The summary of how the Smax and DF were measured for the virtually induced AF is presented in Figure 2. To determine the Smax, the value of the APD_90_ and DI were measured during pacing on the anterior side of the LA from the start of the pacing to 3 beats after the AF induction at each node. The Smax was calculated as the maximum slope of the restitution curve and was defined for all nodes of the LA model. The nonlinear fitting of the APD_90_ and DI was calculated using the following correlation equation (30):


$$\begin{array}{l} y\left(\text{Action potential duration}\right)=y_{0}-A_{1}(1-e^{-\frac{DI}{\tau_{1}}}) \end{array}$$

where *y*_0_ and *A*_1_ are free-fitting variables, and τ_1_ is a time constant. In each patient, we obtained the Smax of each node and calculated the mean Smax value in 10 regions of the LA. Also, we calculated the mean Smax value of a patient based on the Smax value of all nodes.

The DF was defined as the frequency with the highest peak on the magnitude spectrum and was derived from a Fourier transform for 6 s of action potentials at each node (10). After measuring the Smax, the DF was measured during the maintenance of AF at each node for three consecutive time periods: 16–22, 22–28, and 28–34 s. We obtained the value of the DF for all nodes of the LA model. To perform regional LA analysis, the LA was separated into 10 regions per patient depending on a previous study (31). The mean Smax and DF were calculated in each LA region to use for analysis. AF maintenance was determined by assessing the electrogram and 3D activation map. AF defragmentation included AF termination or conversion of AF to atrial tachycardia.

### Computational DF Ablation

To perform virtual ablations, the conduction block was processed by adjusting the diffusion coefficient parameter. The ablated region in the simulation model was considered as the area in which the electrical conduction could not occur. After the virtual CPVI, virtual DF ablation was performed in the highest 5% of the DF area. The highest 5% of the DF area included all nodes that were within 5% of the highest DF value. At conditions of CPVI alone or CPVI with DF ablation, AF was induced by ramp-pacing stimulation for 11.5 s (Figure 1B). After that, we observed the rhythm status for 10 s to investigate AF termination or AF defragmentation, defined as AF termination or conversion to atrial tachycardia.

### Clinical AFCA With DF Ablation

A detailed ablation protocol of CUVIA-AF2 was reported in a previous study (20). In summary, the operator generated an electroanatomical map, including the bipolar voltage map and local activation map, using a multielectrode catheter (AFocus, Abbott, Chicago, Illinois, USA) under the EnSite™ NavX™ system before ablation. Afterward, the computational DF mapping for additional ablation in the virtual DF-guided ablation group was developed using the digital data of the LA substrate map until the operator conducted the CPVI. Also, the operator conducted additional ablation to target DF areas in the virtual DF-guided ablation group, whereas additional DF ablation was not conducted in the empirical PVI group.

An open irrigated-tip catheter or a contract-force sensing ablation catheter was used to perform the CPVI and DF ablations using 3D electroanatomical mapping (EnSite™ NavX™) merged with 3D-spiral CT. The CPVI with a bidirectional block was performed in all the patients in this study. An additional ablation after CPVI was conducted based on the virtual DF mapping in the DF ablation group and at the operator's discretion in the empirical PVI group. We ablated the DF areas located in the CT-merged 3D electroanatomical map using the focal ablation technique. We used 40–50 W of RF energy for 10–15 s in other LA lesions except for the posterior side of the LA or LA appendage. The procedure was completed unless an AF immediately recurred during the 10-min observation period following cardioversion with an isoproterenol infusion (5–10 μg/min depending on ß-blocker use; target sinus heart rate of 120 bpm; AF induction by a ramp pacing cycle length of 120 ms). In the case of mappable AF triggers or premature atrial beats, extra-PV foci were ablated as much as possible.

### Postablation Management and Follow-Up in Clinical Patients

Patients were scheduled to regularly visit an outpatient clinic at 1, 3, 6, and 12 months after AFCA and every 6 months thereafter or whenever symptoms developed. Patients underwent an ECG at every visit. A 24-h Holter monitoring was performed at 3, 6, and 12 months and then every 6 months after the AFCA according to the guideline (1). Whenever the patients experienced symptoms of palpitations, we examined the Holter/event-monitor results to investigate the possibility of an arrhythmia recurrence. We defined an AF/AT recurrence as any episode of AT or AF lasting 30 s or more. Any electrocardiography documentation of an AF recurrence after a 3-month blanking period was classified as clinical recurrence.

### Statistical Analysis

Categorical variables were reported as numbers (percentages). To investigate the normal distribution, continuous variables were tested by the Shapiro–Wilk or the Kolmogorov–Smirnov test. Continuous variables without normal distribution were expressed as medians with interquartile range (IQR), while those with normal distribution were expressed as means ± SD. The proportion of categorical variables was compared among groups using the chi-squared test or Fisher's exact test. Continuous variables without normal distribution were analyzed using the Mann–Whitney *U*-test between the two groups and using the Kruskal–Wallis test among the three groups. Continuous variables with normal distribution were tested using the ANOVA test among the three groups. The correlation between DF and Smax in the computational model and among the clinical patients was assessed by the Spearman test. The Kaplan–Meier analysis with a log-rank test was performed to assess the freedom from AF/AT recurrence after the AFCA among the clinical patients according to ablation strategy. A Cox regression analysis was used to assess the differences in risk for AF/AT recurrence among the three ablation strategies. A *p* < 0.05 was considered statistically significant. All statistical analyses were performed using SPSS (Statistical Package for Social Sciences, Chicago, IL, United States) software for Windows (version 23.0) and the R software [R Core Team (2021). R: A language and environment for statistical computing. R Foundation for Statistical Computing, Vienna, Austria. URL https://www.R-project.org/].

## Results

### Inverse Modest Relationship Between DF and Smax: A Modeling Study

**Figure 4:**
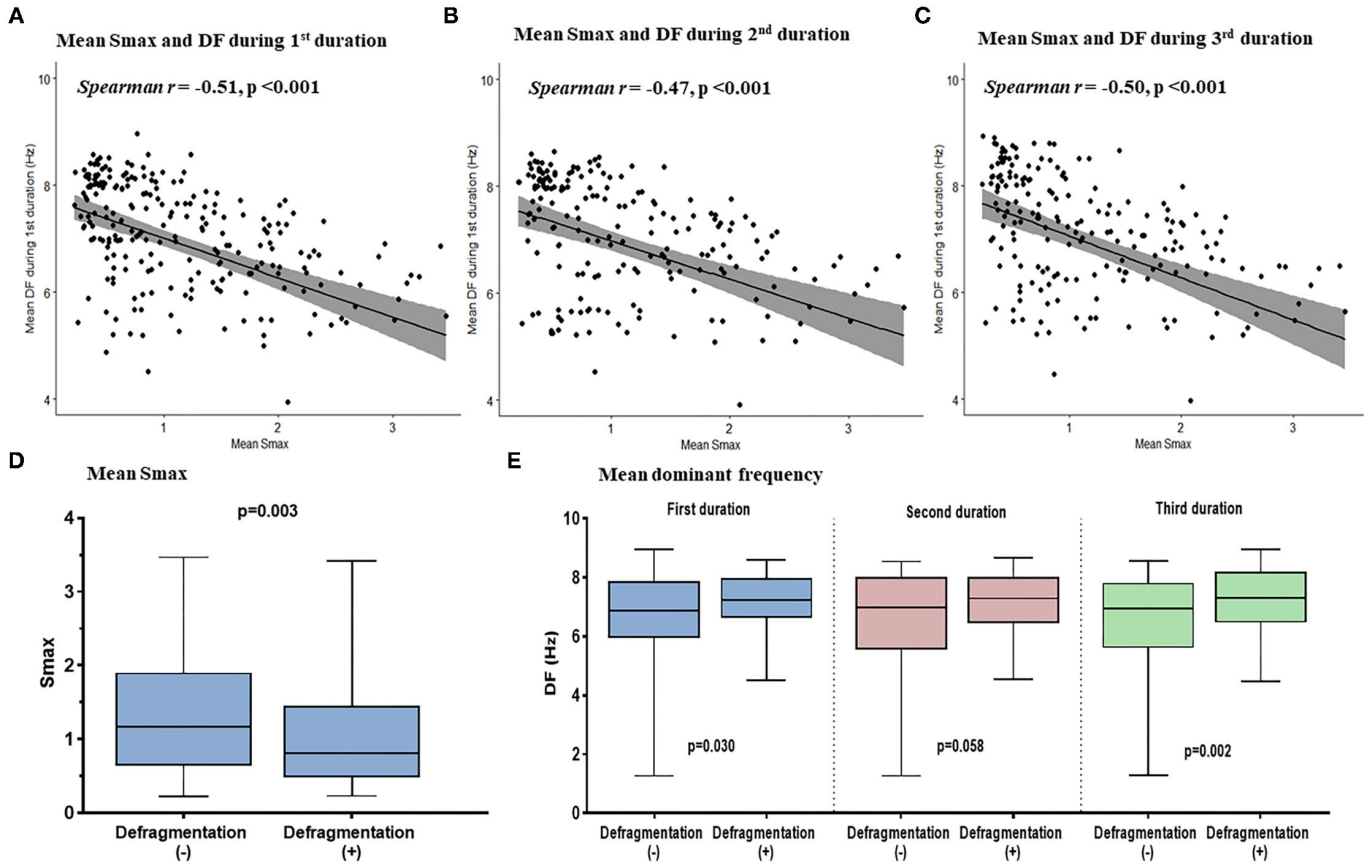
The relationship between the Smax and DF in the computational simulation using 250 regions in 25 patients. The correlation between Smax and DF during 16–22 s **(A)**, 22–28 s **(B)**, and 28–34 s **(C)** after virtual AF induction. Comparing the Smax **(D)** and the DF in three periods **(E)** according to AF defragmentation after virtual DF ablation. A point indicates Smax and DF in a single region of LA. Smax, the maximal slope of action potential restitution curve; DF, dominant frequency; AF, atrial fibrillation; LA, left atrium.

We evaluated the Smax of more than 400,000 nodes of atrial computational modeling during ramp pacing. After AF induction followed by ramp pacing, we evaluated DF at each node over three periods (16–22, 22–28, and 28–34 s). Mean DF and mean Smax were inversely modestly correlated with each other during the three consecutive periods: in the first period (Spearman *r* = −0.51, *p* < 0.001, Figure 4A), the second period (*Spearman r* = −0.47, *p* < 0.001, Figure 4B), and the third period (Spearman *r* = −0.50, *p* < 0.001, Figure 4C). The 13 AF computational models with low Smax (<1) had significantly higher mean DF in the three consecutive AF periods (*p* < 0.001 in three periods, Table 1) compared to the 12 *in-silico* models with high Smax (≥1) with consistency (Table 1).

**Table 1:** Comparing the dominant frequency of three consecutive periods between the low and high Smax conditions in computational simulation.

	**Low smax <1** **(*n* = 13, 130 regions)**	**High smax ≥1** **(*n* = 12, 120 regions)**	***P* value**
Smax	0.61 [0.41, 0.98]	1.52 [0.91, 2.08]	<0.001
Mean DF at 1st duration	7.66 [6.96, 8.13]	6.56 [5.70, 7.16]	<0.001
Mean DF at 2nd duration	7.91 [6.86, 8.23]	6.50 [5.50, 7.15]	<0.001
Mean DF at 3rd duration	7.80 [7.03, 8.37]	6.49 [5.50, 7.14]	<0.001

*Smax, the maximal slope of action potential restitution curve; DF, dominant frequency*.

### Better Anti-AF Effects of DF Ablation at Lower Smax Conditions: A Modeling Study

Additional virtual DF ablation after CPVI showed a significantly higher defragmentation rate of AF compared to CPVI alone (48 vs. 16%, *p* = 0.015, Table 2). The rate was especially high at the condition with Smax <1 and additional virtual DF ablation showed a higher defragmentation rate of AF than of CPVI alone (61.5 vs. 7.7%, *p* = 0.011). However, this was not the case with Smax ≥ 1 (33.3 vs. 25%, *p* > 0.999, Table 2). The episode with AF defragmentation after virtual DF ablation showed a significantly lower Smax [0.81 (IQR: 0.49–1.44) vs. 1.17 (IQR: 0.65, 1.89), *p* = 0.003] compared to the episodes without AF defragmentation (Figure 4D). In the episodes with AF defragmentation after virtual DF ablation, the mean DF value was significantly higher than in those without defragmentation at the first [7.22 Hz (IQR: 6.64–7.95) vs. 6.88 Hz (IRQ: 5.97–7.85), *p* = 0.030, Figure 4E] and third AF periods [7.31 Hz (IQR: 6.50–8.17) vs. 6.94 Hz (IQR: 5.64–7.79), *p* = 0.002, Figure 4E].

**Table 2:** The rate of AF termination or defragmentation after virtual ablation.

	**Overall (*n* = 50)**	**CPVI + DF** **(*n* = 25)**	**CPVI alone (*n* = 25)**	***P* value**
Overall				
AF termination	6 (12%)	4 (16%)	2 (8%)	0.667
AF defragmentation	16 (32%)	12 (48%)	4 (16%)	**0.015**
Low Smax <1	*n* = 26	*n* = 13	*n* = 13	
AF termination	4 (15.4%)	3 (23.1%)	1 (7.7%)	0.593
AF defragmentation	9 (34.6%)	8 (61.5%)	1 (7.7%)	**0.011**
High Smax ≥ 1	*n* = 24	*n* = 12	*n* = 12	
AF termination	2 (8.3%)	1 (8.3%)	1 (8.3%)	>0.999
AF defragmentation	7 (29.2%)	4 (33.3%)	3 (25%)	>0.999

*AF, atrial fibrillation; DF, dominant frequency; CPVI, circumferential pulmonary vein isolation; Smax, the maximal slope of action potential restitution curve. The bold means statistically significant value (p-value < 0.05)*.

### Clinical DF Ablation Effects in the CUVIA-AF2 Trial

**Figure 5:**
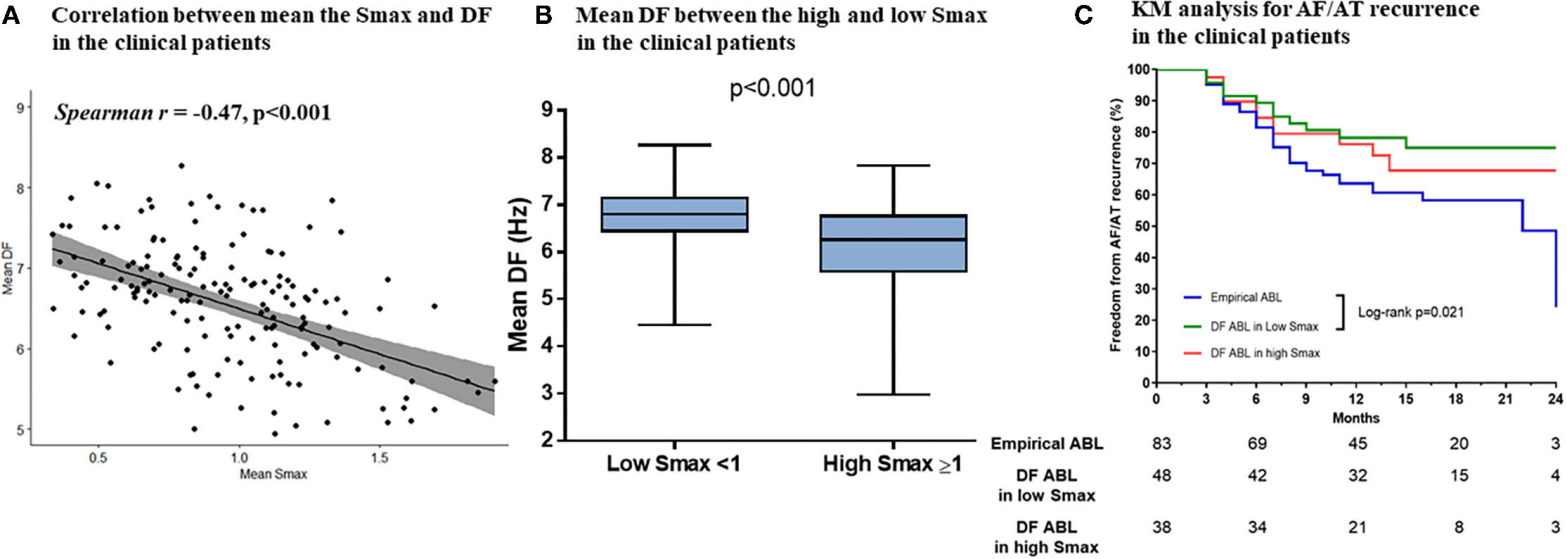
The relationship between the Smax and DF in the clinical patients from the CUVIA AF2 population. The correlation between the Smax and DF in the clinical patients **(A)**. Comparing the DF between low Smax (<1) and high Smax (≥1) conditions in the clinical patients **(B)**. Kaplan–Meier (KM) analysis for clinical AF/AT recurrence among three ablation groups in the clinical patients **(C)**. Smax, the maximal slope of action potential restitution curve; DF, dominant frequency; AF, atrial fibrillation; AT, atrial tachycardia; ABL, ablation.

Baseline characteristics of included patients in the CUVIA-AF2 trial (20) are summarized in Supplementary Table 1. There is no significant difference in clinical parameters, namely, age, sex, and echocardiographic parameters between DF ablation with the low Smax (<1) group and DF ablation with the high Smax (≥1) patient group. Consistent with the virtual modeling studies, there was an inverse modest correlation between Smax and DF (Spearman *r* = −0.47, *p* < 0.001, Figure 5A). DF was significantly higher in low Smax (<1) patients than in high Smax (≥1) patients [6.80 Hz (IQR: 6.45–7.15) vs. 6.27 Hz (IQR: 5.60–6.78), *p* < 0.001, Figure 5B].

Table 3 compares baseline characteristics among the empirical ablation (*n* = 83), DF ablation at Smax <1 (*n* = 48), and DF ablation at Smax ≥ 1 (*n* = 38) groups in the CUVIA-AF2 trial (20). The rhythm outcome of clinical AF ablation was significantly superior in the DF ablation at Smax <1 group than in the empirical ablation group (log-rank *p* = 0.021, Figure 5C) but not in the DF ablation at Smax ≥ 1 group (log-rank *p* = 0.177). In multivariate Cox regression analysis, DF ablation in addition to CPVI at Smax <1 was independently associated with better rhythm outcome after persistent AF ablation [adjusted HR 0.45, 95% CI (0.222–0.891), *p* = 0.022, Table 4].

**Table 3:** Baseline characteristics among the three ablation groups in the clinical patients.

	**Empirical PVI (*n* = 83)**	**Low Smax <1** **DF ablation** **(*n* = 48)**	**High Smax ≥1 DF ablation (*n* = 38)**	***P* value**
Age, years	61 (52.5, 69)	58.5 (51, 64)	61 (55, 66)	0.475
Male, *n*	53 (63.9%)	36 (75%)	31 (81.6%)	0.106
AF duration, months	24 (12, 60)	17 (9, 48)	26 (10, 48)	0.320
CHF	18 (21.7%)	12 (25%)	10 (26.3%)	0.829
Hypertension	42 (50.6%)	27 (56.2%)	23 (60.5%)	0.570
Diabetes mellitus	20 (24.1%)	9 (18.8%)	9 (23.7%)	0.764
Stroke or TIA	14 (16.9%)	7 (14.6%)	8 (21.1%)	0.728
Vascular disease	4 (4.8%)	5 (10.4%)	3 (7.9%)	0.474
CHA2DS2VASc score	2 (1, 3)	2 (1, 2)	2 (1, 3)	0.727
LA dimension	44.7 ± 5.5	44.3 ± 6.0	45.7 ± 5.0	0.454
LA volume index	39.0 (31.4, 49.0)	35.5 (28.0, 44.1)	42.0 (32.9, 48.8)	0.254
LV ejection fraction	62 (57, 65)	61 (56, 67)	60 (55, 65)	0.433
E/Em	9.0 (7.6, 11.9)	8.8 (7.5, 11.2)	8.7 (7.3, 12.3)	0.532

*PVI, pulmonary vein isolation; Smax, the maximal slope of action potential restitution curve; DF, dominant frequency; AF, atrial fibrillation; CHF, congestive heart failure; TIA, transient ischemic attack; LA, left atrium; LV, left ventricle; E/Em, mitral inflow velocity/mitral annulus tissue velocity*.

**Table 4:** The Cox regression analysis for AF/AT recurrence in the clinical patients.

**Variables**	**Univariate analysis**		**Multivariate analysis**	
	**HR (95% CI)**	***P* value**	**HR (95% CI)**	***P* value**
Age	0.995 (0.972–1.019)	0.689	0.993 (0.969–1.017)	0.548
Male	0.984 (0.562–1.724)	0.956	1.100 (0.602–2.009)	0.756
AF duration	1.002 (0.996–1.007)	0.595		
Congestive heart failure	0.754 (0.397–1.432)	0.389		
Hypertension	1.077 (0.633–1.833)	0.784		
Diabetes mellitus	1.153 (0.630–2.109)	0.644		
Stroke or TIA	1.817 (0.991–3.331)	0.053		
Vascular disease	0.723 (0.226–2.317)	0.585		
CHA_2_DS_2_VASc score	1.045 (0.886–1.233)	0.601		
LA dimension	1.011 (0.963–1.061)	0.664	1.015 (0.966–1.067)	0.559
LV ejection fraction	1.006 (0.975–1.038)	0.714		
E/Em	0.989 (0.933-1.048)	0.707		
Empirical PVI ablation	Reference		Reference	
DF ablation in low Smax (<1)	0.468 (0.237–0.926)	**0.029**	0.445 (0.222–0.891)	**0.022**
DF ablation in high Smax (≥1)	0.644 (0.327–1.269)	0.204	0.557 (0.272–1.138)	0.108

*AT, atrial tachycardia, other abbreviations were shown in Table 3. The bold means statistically significant value (p-value < 0.05)*.

## Discussion

### Main Findings

In this study, we demonstrated an inverse modest relationship between Smax and DF in both computational simulation and clinical study. There was a trend for high DF value in low Smax conditions. Virtual DF ablation in addition to CPVI showed a higher defragmentation rate of AF compared to CPVI alone, especially with Smax <1. In the CUVIA-AF2 clinical trial, clinical DF ablation at the condition with Smax <1 showed better rhythm outcomes compared to empirical PVI, consistent with the computational modeling studies.

### Extra-PV Ablations in Persistent AF

Uniform additional ablations beyond CPVI that do not take into account individual electroanatomical remodeling of LA in all patients with persistent AF can no longer be considered a proper strategy to improve ablation outcomes (5). Depending on personalized substrate remodeling or the mechanisms of AF, tailored ablation strategies, namely, MRI-detected low voltage ablation (32), rotor or focal source ablation (33), and DF mapping-guided ablation (34), have been developed to improve the rhythm outcomes of AFCA. However, because their efficacy for rhythm outcomes is lacking and controversial, additional ablation strategies as the next step after CPVI are not definitely recommended in the current guideline (1), Rotor ablation using a multipolar basket catheter revealed an inconsistent rhythm outcome of AFCA (6, 7), with the limitation of spatial resolution of the rotational activation map (8). CFAE ablation also showed the inconsistent outcome of AFCA (35, 36) depending on the mapping systems and a definition of CFAEs in those studies (37). There was a difference in the results of the DF ablation between the RADAR-AF trial (34) and CUVIA AF2 trial (12), which had different mapping methods for targeting the DF.

In the current situation where the superior outcome of DF mapping-guided ablation is not solid (13). this study demonstrated that ablation for targeting high DF areas among patients with low Smax (<1) showed better rhythm outcomes in the computational simulation and clinical patients.

### AF Drivers and Passive Wave-Break

The focal AF drivers and multiple wavelets hypothesis are considered the main mechanisms of AF maintenance. The focal AF drivers explain that a spiral-wave reentry around phase singularity produces focal source drivers or rotors that maintain AF (38). The multiple wavelets hypothesis explains that multiple independent wavelets propagate and are disrupted into wandering daughter wavelets, which sustain fibrillatory waves (39). High DF represented the location of rotors or focal source AF drivers (9), and the value of Smax reflected the degree of wave-break to maintain fibrillation waves (40), In particular, a high Smax (>1) was related to a high probability of wave-breakup and fibrillatory waves (40). Therefore, we considered DF to be an indicator of the AF mechanism related to focal AF drivers and Smax to be an indicator of passive wave-break. The priority mechanism of AF maintenance can be variable depending on the tissue condition and electrical characteristics. In flat action potential restitution with a low tendency of wave-break, focal source AF drivers might sustain fibrillation waves (41).

### AF at the Condition of Smax <1.0

APD restitution is the myocardial cellular characteristic that reflects the change of APD to the constant DI change (18, 19). Therefore, the oscillation or dynamic heterogeneity of APD becomes more significant with short-coupled premature beats or rapid pacing at the condition of the higher maximal slope of APD restitution, Smax (20, 42). Smax at the myocardial tissue level represents the vulnerability of fibrillatory wave-break because differences in the refractoriness among adjacent cardiomyocytes result in local partial conduction block leading to the re-entrant wavefronts (17, 40). Mathematically, continuous and passive wave-break of spiral waves are easily maintained at Smax > 1 condition because extra beats do not converge to the original restitution curve (20, 42). In contrast, myocardial condition with Smax <1 is hard to maintain AF after induction. However, there are cases where AF is maintained even in the low Smax state. In this situation, the dominant rotor or driver exists somewhere in the atrium as an engine of AF maintenance. In this study, we demonstrated the inverse relationship between Smax and DF, an index of the active driver. In addition, DF ablation was more effective in AF rhythm control at the condition of Smax <1 by analyzing the results of the CUVIA-AF2 clinical study (12).

### Computational Modeling Reflects Realistic AF Wave Dynamics and Future Direction

Computational modeling has been reported to investigate AF mechanisms (13). Recently, MRI-based computational AF modeling was based on personalized atrial anatomy and fibrosis (11). In contrast, the current AF model included personalized electrophysiology in addition to anatomy and fibrosis (23). Therefore, this modeling reflected both AF mechanisms, namely, Smax and DF, and showed better rhythm outcomes of AFCA in clinical settings. However, our AF model needs invasive parameters and fast on-site computation to develop a realistic and physiological simulation during an ablation procedure. In addition to the parameters used in this study, the location of the AF drivers was determined according to the gradient of the atrial wall thickness (43, 44). The cardiac autonomic activity changed the intracellular calcium concentration and APD, leading to the triggering and perpetuation of AF (45–47). The cardiac adipose tissue was associated with inflammation and atrial fibrosis (48). The mechanoelectrical feedback affected the dynamics and behavior of the spiral waves (49, 50). Because all those parameters could affect the atrial electroanatomical remodeling leading to a change in the potential pathophysiology and mechanisms of AF, the location and dynamics of the focal AF source drivers in the simulation model could differ if considering those parameters as compared to the current simulation model.

### Limitations

This study had several limitations in computational simulation and clinical settings. First, biatrial modeling, including interatrial conduction, was not reflected in this modeling study. Second, there was a possibility that atrial fibrosis could not be measured precisely by bipolar voltage because of differences in mapping systems, catheters, and operators compared to those of MRI-detected atrial fibrosis. Third, because we included a small number of patients in the computational simulation and CUVIA AF 2 cohort, the results derived from the virtual simulation and clinical ablation cannot be generalized to all patients with AF. Fourth, this study was an observational cohort study from a single center that included patients who were referred for AF ablation. Fifth, we are not sure DF ablation in addition to CPVI can be applied to patients with high Smax. Sixth, because the definition and cut-off value for detecting focal source drivers cannot always be the same in all clinical settings (37), the current AF modeling may not always find the target for ablation using the Smax and DF. Seventh, classical restitution properties are cellular electrophysiological properties recorded by single-cell membrane potential. For that reason, our computational modeling was designed to implement a node size that was relatively similar to the size of the cardiomyocytes so that the restitution property could be applied to each node. Eighth, although we measured the DF for 6 s three times sequentially (total 18 s), the DF may be spatiotemporally unstable depending on the tissue condition, especially regarding the conduction velocity (51–53). Ninth, discordant alternans induce spatial dispersion of the refractoriness, which causes a conduction block at some cardiomyocytes leading to fibrillation waves (54, 55). but was not reflected in the current computational model.

## Conclusion

In this computational AF model, there was an inverse modest correlation between Smax and DF. The additional DF ablation under the low Smax (<1) condition showed beneficial rhythm outcomes in computational simulation and clinical settings. These results suggest that both the focal source driver and passive wave-break are needed to perpetuate AF and should be considered simultaneously to improve ablation outcomes.

## Data Availability Statement

The original contributions presented in the study are included in the article/Supplementary Material, further inquiries can be directed to the corresponding author. The datasets generated and/or analyzed during this study are not publicly available because of the sensitive nature of the data; requests to access the dataset from qualified researchers trained in human subject confidentiality protocols may be sent to the corresponding author.

## Ethics Statement

The studies involving human participants were reviewed and approved by the Institutional Review Board of the Yonsei University Health System. The patients/participants provided their written informed consent to participate in this study.

## Author Contributions

J-WP, BL, and H-NP designed the current study, performed data analysis, and wrote the manuscript. BL, IH, and O-SK contributed to the customized software generation. HY, T-HK, J-SU, BJ, and M-HL contributed to acquiring the patients' clinical data. J-WP and H-NP interpreted and discussed the results. All persons designated as authors qualify for authorship and all those who qualify for authorship are listed. All authors approved the final version of the manuscript.

## Funding

This study was supported by grants (HI19C0114) and (H21C0011) from the Ministry of Health and Welfare and grants (NRF-2020R1A2B01001695) and (NRF-2019R1C1C1009075 to BL) from the Basic Science Research Program run by the National Research Foundation of Korea (NRF), which is funded by the Ministry of Science, ICT, and Future Planning (MSIP).

## Conflict of Interest

The authors declare that the research was conducted in the absence of any commercial or financial relationships that could be construed as a potential conflict of interest.

## Publisher's Note

All claims expressed in this article are solely those of the authors and do not necessarily represent those of their affiliated organizations, or those of the publisher, the editors and the reviewers. Any product that may be evaluated in this article, or claim that may be made by its manufacturer, is not guaranteed or endorsed by the publisher.

## Supplementary Material

Supplementary Figure 1 The example of the APD restitution curve with a high Smax (≥1) in one node **(A)** and a low Smax (<1) in another node **(B)**. APD, action potential duration; Smax, the maximal slope of action potential restitution curve; DI, diastolic interval.

Supplementary Figure 2 The enrollment of the clinical patients from the CUVIA AF2 trial. AF, atrial fibrillation; PVI, pulmonary vein isolation; DF, dominant frequency; Smax, the maximal slope of action potential restitution curve.

Supplementary Table 1 Baseline characteristics in the clinical patients with high and low Smax. Abbreviations were shown in Table 3.
