# Supplementary figures and images for: Restitution Slope Affects the Outcome of Dominant Frequency Ablation in Persistent Atrial Fibrillation: CUVIA-AF2 Post-Hoc Analysis Based on Computational Modeling Study
Source: Front Cardiovasc Med. 2022 Mar 3;9:838646. doi: 10.3389/fcvm.2022.838646 (PMC8927985; doi:10.3389/fcvm.2022.838646)

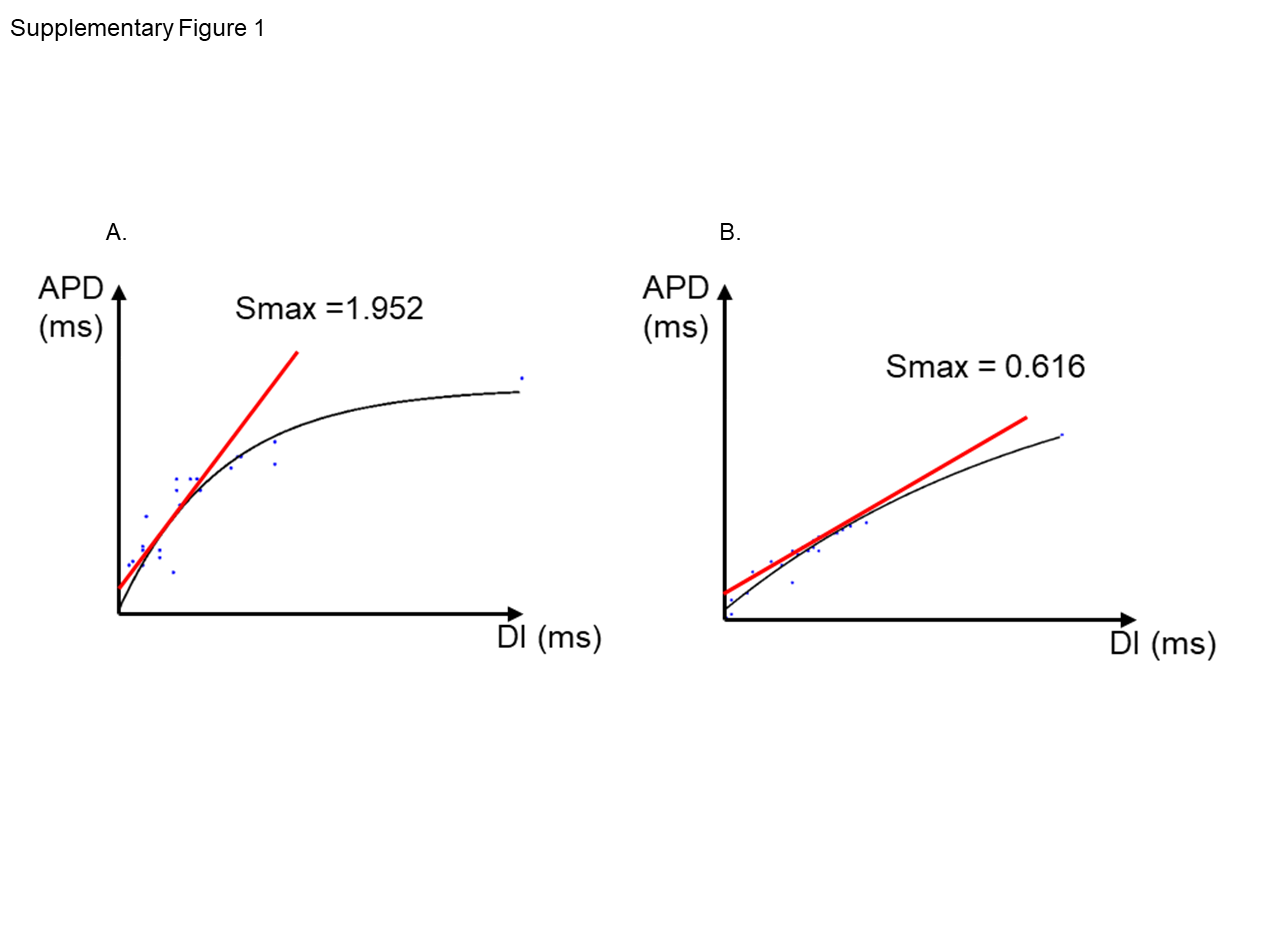

Supplement: Supplementary Figure 1 — The example of the APD restitution curve with a high Smax (≥1) in one node (A) and a low Smax (<1) in another node (B). APD, action potential duration; Smax, the maximal slope of action potential restitution curve; DI, diastolic interval. [file Image_1.tif]

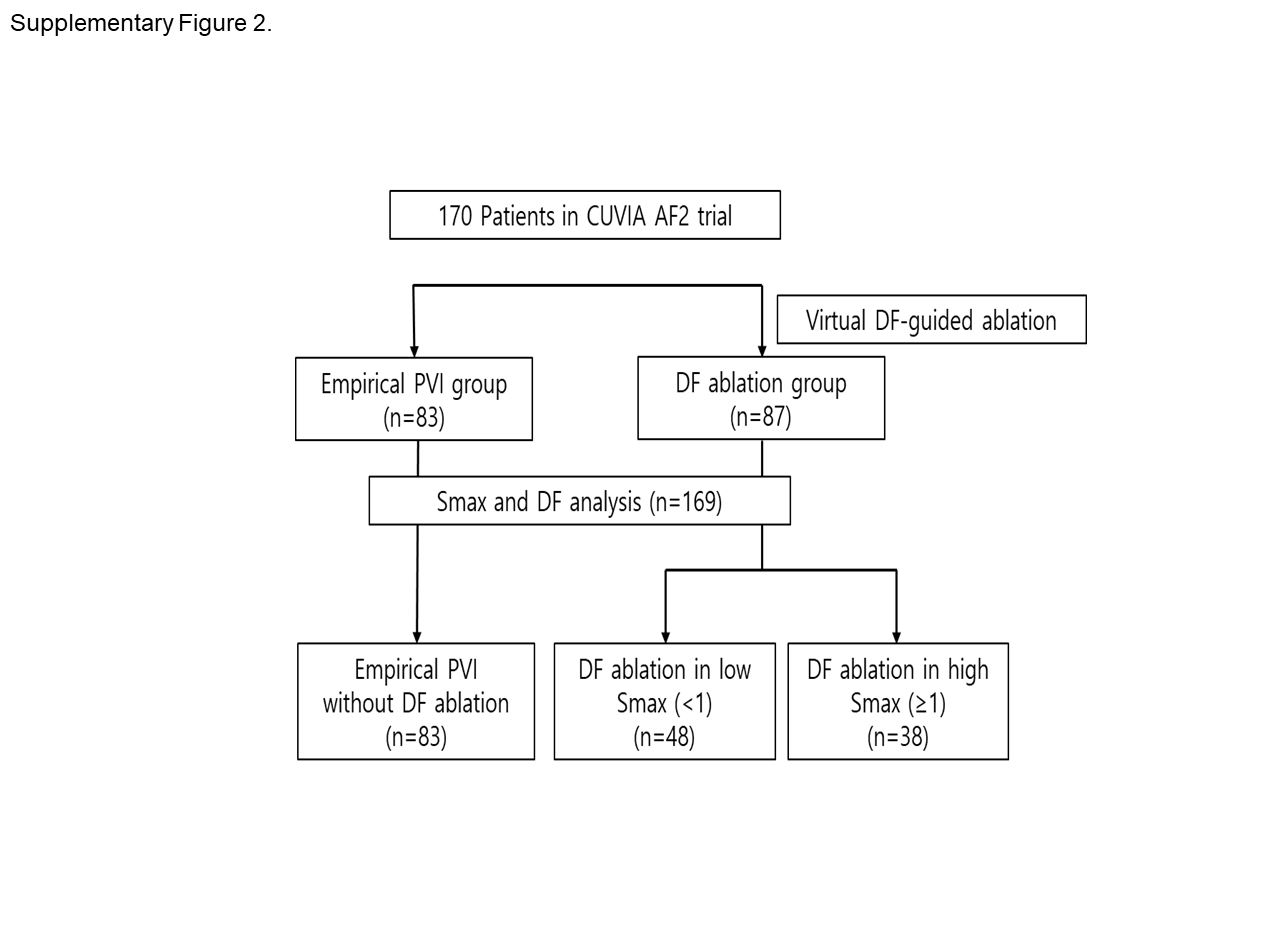

Supplement: Supplementary Figure 2 — The enrollment of the clinical patients from the CUVIA AF2 trial. AF, atrial fibrillation; PVI, pulmonary vein isolation; DF, dominant frequency; Smax, the maximal slope of action potential restitution curve. [file Image_2.tif]
