# Supplementary material for: Restitution Slope Affects the Outcome of Dominant Frequency Ablation in Persistent Atrial Fibrillation: CUVIA-AF2 Post-Hoc Analysis Based on Computational Modeling Study
Source: Front Cardiovasc Med. 2022 Mar 3;9:838646. doi: 10.3389/fcvm.2022.838646 (PMC8927985; doi:10.3389/fcvm.2022.838646)
Supplement: Supplementary Table 1 — Baseline characteristics in the clinical patients with high and low Smax. Abbreviations were shown in Table 3. [file Table_1.docx]

**Supplementary Table 1. Baseline characteristics in the clinical patients with high and low Smax**

|  | **Overall patients**  **(n=170)** | **Low Smax<1**  **(n=92)** | **High Smax≥1**  **(n=75)** | **P-value** |
| --- | --- | --- | --- | --- |
| Age, years | 60 (53, 66) | 59 (51, 66) | 61 (55, 66) | 0.220 |
| Male, n | 120 (70.6%) | 62 (67.4%) | 56 (74.7%) | 0.392 |
| AF duration, months | 24 (10, 48) | 24 (11, 48) | 24 (10, 48) | 0.754 |
| Congestive her failure | 40 (23.5%) | 22 (23.9%) | 18 (24&) | >0.999 |
| Hypertension | 93 (54.7%) | 50 (54.3%) | 40 (53.3%) | >0.999 |
| Diabetes mellitus | 39 (22.9%) | 21 (22.8%) | 16 (21.3%) | 0.965 |
| Stroke or TIA | 30 (17.6%) | 12 (13%) | 17 (22.7%) | 0.153 |
| Vascular disease | 13 (7.6%) | 5 (5.4%) | 7 (9.3%) | 0.503 |
| CHA2DS2VASc score | 2 (1, 3) | 2 (1, 3) | 2 (1, 3) | 0.542 |
| LA dimension | 45.0 (41.5, 49.0) | 44.3 ± 6.0 | 45.6 ± 4.8 | 0.154 |
| LA volume index | 38.9 (31.0, 47.8) | 36.1 (29.0, 46.4) | 40.2 (32.2, 49.0) | 0.106 |
| LV ejection fraction | 61 (57, 65) | 62 (57, 66.5) | 60 (57.0, 65.0) | 0.350 |
| E/Em | 9.0 (7.5, 11.6) | 8.8 (7.6, 11.4) | 9.2 (7.3, 11.8) | 0.566 |

Abbreviations were shown in table 3.
